# New Sydenham Society’s Publications

**Published:** 1868-06

**Authors:** 


### New Sydenham Society's Publications.

Having made arrangements with the Hon. Local Secretary, Richard J. Dunglison, M. D., by and with the approval of the Society's Agent in London, to act as Agents in the United States for the publications of the New Sydenham Society, announce that they are now prepared to receive subscriptions for the year 1868, at *Ten Dollars*, payable in currency and invariably in advance, and to furnish any of the previous years at the same rate and on the same terms.

The Practical Character and Permanent Value of these publications and the very low price at which they are furnished, commend them to the favorable attention of the Medical Profession in the United States.

Four Volumes will be issued for 1868. The following works are now in preparation:

A Collected Edition of the Works of Dr. Addison; with Preface, Notes, Portrait and numerous Lithographs.

A Descriptive Catalogue of the Portraits already issued in the Atlas of Skin Diseases.

The Eighth Fasciculus of the Atlas of Skin Diseases.

The Second Volume of Hebra on Diseases of the Skin.

The Second Volume of Trousseau's Clinical Medicine.

The First Volume of Lancereau's Treatise on Syphilis.

Subscribers at a distance can have the Volumes as they appear, forwarded to them by mail, upon remitting to Lindsay & Blakiston, Medical Publishers and Booksellers, No. 25 South Sixth street, Philadelphia, in addition to the subscription, 50 cents per volume for the postage, which must be paid in advance.

To Subscribers of the London Lancet.—The subscriber begs to inform his patrons, that he has entered into an arrangement with the London publishers, for the advanced sheets of The Lancet. The work will appear on the first of the month hereafter.

*All subscriptions should be sent in at once*, so as to enable the Agent to meet his enhanced home and foreign expenses.

William C. Herald,

Agent for the London Lancet.

